# Local conditions have greater influence than provenance on sugar maple (*Acer saccharum* Marsh.) frost hardiness at its northern range limit

**DOI:** 10.1093/treephys/tpae167

**Published:** 2024-12-27

**Authors:** Claudio Mura, Guillaume Charrier, Valentina Buttò, Sylvain Delagrange, Yann Surget-Groba, Patricia Raymond, Sergio Rossi, Annie Deslauriers

**Affiliations:** Université du Québec à Chicoutimi, Département de Sciences Fondamentales, laboratoire écosystèmes terrestres boréaux (EcoTer), 555 boulevard de l'Université, G7H 2B1 Chicoutimi, QC, Canada; Université Clermont Auvergne-INRAE, UMR Integrative Physics and Physiology of Trees in Fluctuating Environments (PIAF), 5 chemin de Beaulieu, 63000 Clermont-Ferrand, France; Université du Québec en Abitibi-Témiscamingue, Institut de recherche sur les forêts (IRF), 445 boulevard de l'Université, J9X 5E4 Rouyn-Noranda, QC, Canada; Université du Québec en Outaouais, Institut des sciences de la forêt tempérée, 58 rue Principale, J0V 1V0 Ripon, QC, Canada; Université du Québec en Outaouais, Institut des sciences de la forêt tempérée, 58 rue Principale, J0V 1V0 Ripon, QC, Canada; Ministère des Ressources naturelles et des Forêts (MRNF), Direction de recherche forestière, 2700 rue Einstein, G1P 3W8 Québec, QC, Canada; Université du Québec à Chicoutimi, Département de Sciences Fondamentales, laboratoire écosystèmes terrestres boréaux (EcoTer), 555 boulevard de l'Université, G7H 2B1 Chicoutimi, QC, Canada; Université du Québec à Chicoutimi, Département de Sciences Fondamentales, laboratoire écosystèmes terrestres boréaux (EcoTer), 555 boulevard de l'Université, G7H 2B1 Chicoutimi, QC, Canada

**Keywords:** cold resistance, frost vulnerability, phenology, populations

## Abstract

In temperate and boreal ecosystems, trees undergo dormancy to avoid cold temperatures during the unfavorable season. This phase includes changes in frost hardiness, which is minimal during the growing season and reaches its maximum in winter. Quantifying frost hardiness is important to assess the frost risk and shifts of species distribution under a changing climate. We investigate the effect of local conditions and intra-specific variation on frost hardiness in sugar maple (*Acer saccharum* Marsh.). Seedlings belonging to seven provenances from the northern area of the species’ range were planted at two sites in Quebec, Canada. LT_50_, i.e. the lethal temperature for 50% of the cells, was measured monthly with the relative electrolyte leakage method on branches and buds from September 2021 to July 2022. LT_50_ varied between −4 °C in summer (July) and −68 °C in winter (February). Autumnal acclimation rates (September to early December) and mid-winter frost hardiness (December to early March) were similar in both sites. Samples in the southern site deacclimated faster than in the northern site between March and July because of a warmer and earlier spring. No difference in frost hardiness was detected between provenances. Our results suggest that the frost hardiness trait is similar within the northern part of the sugar maple distribution, with local weather conditions having a greater influence than provenance. We demonstrate that LT_50_ in sugar maple can exceed −55 °C, far below the minimum temperatures occurring in winter at the northern limit of the species. In order to minimize the risk of damage from extreme frost events exceeding tree frost hardiness, a careful evaluation of site characteristics is more important than provenance selection. Other factors should also be considered within the context of changing climate, in particular, the phenology of maple and avoidance of late frost in spring.

## Introduction

In cold environments, trees have developed the ability to increase their frost hardiness to endure winter conditions. Temperatures exceeding the plant’s vulnerability to frost can cause severe tissue damage and death (Sakai and Larcher 1987). Frost damage occurs when cold temperatures induce ice formation within plant tissues, which can lead to rupture of the plasma membrane and cell death (Uemura et al. 2006). Plants reduce the risk of frost damage by synchronizing their growing season with the warmer period of the year through phenological adjustments (frost avoidance) and increasing the frost hardiness of overwintering organs through physiological adjustments (frost tolerance) (Charrier et al. 2015). However, questions remain regarding the intra-specific differences in frost hardiness resulting from local populations and their implications for forest adaptation to climate change.

At the cellular level, trees can increase their frost hardiness by lowering the freezing point of the cytosol and inducing ice formation outside of the cell to avoid intracellular ice formation and membrane rupturing. This is partially accomplished by physiological adjustments, for example, reducing the water content and increasing the concentration of soluble sugars in the cells (Baffoin et al. 2021, Deslauriers et al. 2021). Frost hardiness therefore changes during the year, being at a minimum during the growing season and at a maximum in mid-winter (Lang et al. 1987, Sakai and Larcher 1987). For example, a study on boreal *Vaccinium* spp. found that frost resistance, quantified as the temperature inducing lethal damage to 50% of the cells, varied between −5 °C during the growing season and −67 °C during winter (Deslauriers et al. 2021).

Frost hardiness dynamics are closely linked with the phenological cycle of growth and dormancy (Leinonen 1996, Vitasse et al. 2014*b*). In mid-summer, trees stop radial growth and form buds to protect the meristems (Rohde and Bhalerao 2007, Buttò et al. 2021). In autumn, colder temperatures and shortening photoperiod induce dormancy (Way 2011, Hamilton et al. 2016). Initially, trees enter the endodormancy phase, which is internally regulated by growth inhibitors and requires exposure to cold temperatures (i.e. chilling) to be released (Lang et al. 1987, Chuine et al. 2016). During endodormancy, frost hardiness increases in overwintering organs (i.e. acclimation) until reaching a peak in winter (Sakai and Larcher 1987, Vitasse et al. 2014*b*). During autumn and winter, exposure to cold temperatures releases endodormancy, and the tree enters the ecodormancy phase, which is mainly controlled by temperature (Junttila 2007, Charrier et al. 2015, Delpierre et al. 2016) with a minor effect of photoperiod in some species (Way and Montgomery 2015, Flynn and Wolkovich 2018, Malyshev et al. 2018). The gradual rise in temperature during spring, defined as forcing units, releases ecodormancy and triggers a decrease in frost hardiness (deacclimation) until budbreak and the resumption of growth (Hänninen 1990, Charrier et al. 2011, Kovaleski et al. 2018).

Frost hardiness is a critical trait for tree survival in cold climates and is considered one of the main factors limiting poleward expansion of species range (Sakai and Larcher 1987, Inouye 2000). Accordingly, the hardiness zones commonly used to define climate suitability for plant species in North America are based on absolute minimum temperatures (Daly et al. 2012). In a study on 27 native and exotic species growing in a common garden in central Europe, Kreyling et al. (2015) found that tree frost hardiness correlated to the climate at the species’ origin, particularly the minimum temperature of the coldest month. Some studies have pointed out the importance of off-season late frosts as a major driver of natural selection and range expansion (Lenz et al. 2013, Körner et al. 2016). However, analyses of frost hardiness are often time-consuming and labor-intensive, which limits their application to special or localized cases (Burr et al. 2001). For this reason, field data quantifying tree frost hardiness during acclimation and deacclimation are still missing for many species.

Under climate change, the habitats of many species are expected to shift poleward or upwards toward sites submitted to colder conditions (McKenney et al. 2007, Boisvert-Marsh and Périé 2014). While winters will generally become warmer, the weather is becoming more variable and less predictable, making frost events likely even under global warming scenarios (Screen and Simmonds 2013, Marquis et al. 2022). Accordingly, warming events during winter or spring can induce deacclimation and lead to frost damage when the temperatures again fall below 0 °C (Augspurger 2013, Vitasse et al. 2014*b*, Zohner et al. 2020). Even if not lethal, frost events can have critical impacts on tree growth and competitiveness. Montwé et al. (2018) showed that a frost event happening 20 years after planting significantly affected the growth in a *Pinus contorta* (Dougl. ex. Loud) common garden, which remained low in subsequent years. Reduced growth and performance of trees and saplings can also hinder the establishment and long-term survival of new cohorts, thus limiting poleward or upward expansion of tree species range (Winder et al. 2011, Kreyling et al. 2015). Frost hardiness is, therefore, an important aspect of predicting the frequency of frost damages, the potential range expansion of trees, and the consequent evolution of forest structure.

Different populations of the same species can differ in their frost hardiness and phenology. A study by Charrier et al. (2011) found that *Juglans regia* (L.) genotypes artificially selected for fruit production reached lower frost hardiness (lethal temperature of −28 °C) than *J. regia × nigra* hybrids selected for wood production (lethal temperature of −35 °C). Intra-specific differences can also occur naturally across latitudinal ranges, where populations from colder climates can exhibit greater frost tolerance and winter survival (Vitasse et al. 2014*b*). This trend has been observed both in conifers (Viveros-Viveros et al. 2009, Kreyling et al. 2012) and angiosperms (Li et al. 2003, Kreyling et al. 2014). However, other studies suggested that intra-specific differences in frost hardiness are limited to one phase of the frost hardiness cycle. Beuker et al. (1998) found differences in frost hardiness during autumnal acclimation but not during winter and deacclimation in *Pinus sylvestris* (L.) and *Picea abies* ([L.] Karst). Instead, Charrier et al. (2011) found differences between *J. regia* genotypes in deacclimation but not during acclimation or midwinter. Understanding species-specific drivers of variations in frost hardiness can provide relevant information to guide tree provenance selection under climate change (Hänninen 2006, Rammig et al. 2010). This is particularly true in cases where tree species and provenances are artificially transferred poleward, i.e. in assisted migration (Ste-Marie et al. 2011). Assisted migration is a tool to promote forest resilience in the face of a rapidly changing climate (Twardek et al. 2023). However, transferring trees poleward begs the question of whether they will be able to survive in colder climates, especially at the sapling stage (Pedlar et al. 2011, Aubin et al. 2016). Quantifying intra-specific differences in frost hardiness can, therefore, help the selection of provenances for assisted migration, increasing the chances of success.

**Figure 1:**
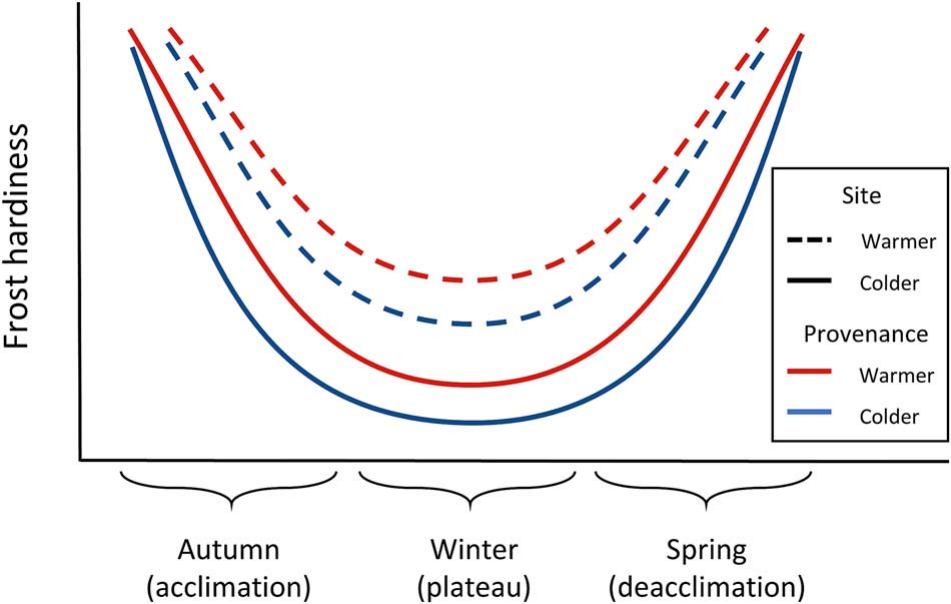
Hypotheses tested for the effect of provenance and site on frost hardiness in sugar maple. We expect samples in the warmer site (dashed lines) to reach lower frost hardiness than in the northern site (solid lines). Within each site, we expect that provenances from colder areas will show faster acclimation but slower deacclimation than provenances from warmer areas. In this study, we use the temperature causing lethal damage to the tree (LT_50_) as an indicator of frost hardiness at a given time.

In this study, we measured frost hardiness in sugar maple (*Acer saccharum* Marsh.) seedlings from seven provenances located in the northern portion of the species distribution and growing in two sites in the Quebec province, Canada. We aimed to quantify intra-specific variability in frost hardiness by comparing the effect of provenance and local environmental conditions. We raised the hypothesis that local weather influences frost hardiness, with seedlings in the colder site showing earlier and faster acclimation in autumn, higher maximum frost hardiness during winter, and later and slower deacclimation in spring, compared with the southern site (Figure 1). We also expect that climatic conditions at the provenance origin influence frost dynamics, with seedlings from colder provenances showing earlier and faster acclimation in autumn, higher frost hardiness, and later, slower deacclimation in spring compared with warmer provenances (Figure 1).

## Materials and methods

### Provenance selection

**Figure 2:**
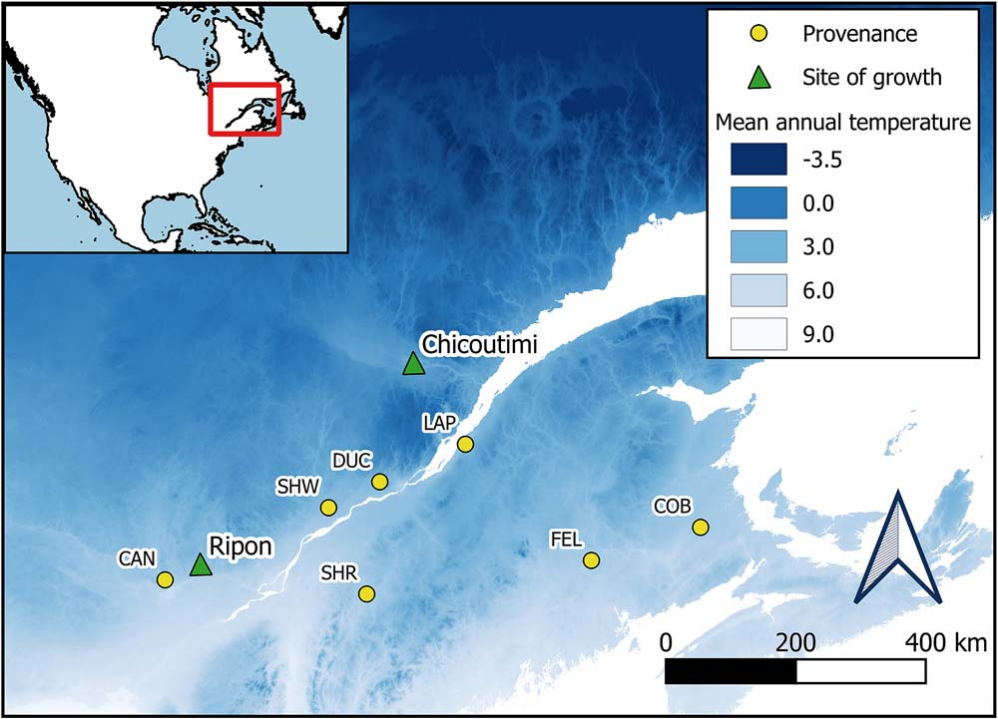
Locations of the seven sugar maple provenances studied (points) and the two sites where seedlings were grown and sampled (triangles).

The plant material for this study consisted of 2-year-old seedlings of sugar maple (*Acer saccharum* Marsh.) belonging to seven commercial provenances from Eastern Canada (Table 1, Figure 2). We focused on this study area, which corresponds to the northern portion of sugar maple’s range because a comparison of frost hardiness for different provenances in this zone is currently missing from the literature. Seeds were collected on single mother trees for Duchesnay, Coy Brook and First Eel Lake provenances (abbreviated as DUC, COB and FEL, respectively) by the National Tree Seed Center (Natural Resources Canada, Fredericton, Canada). The provenances Shawinigan, La Pocatière, Cantley and Sherbrooke (abbreviated as SHW, LAP, CAN and SHR, respectively) originated from seeds collected at stand level by the Ministère des Ressources Naturelles et des Forêts du Québec, Canada. All stands of seed collection are natural (i.e. no artificial selection or tree breeding) and are considered representative of the area of provenance.

**Table 1:** Characteristics of the seven sugar maple provenances studied in this work. Mother tree indicates whether the seeds were collected on a single tree or on multiple trees for each seed lot. Climate data are relative to the 1980–2010 period (source: BioSIM).

Name	ID	Latitude	Longitude	Elevation (m a.s.l.)	Mother tree	Annual temperature (°C)	Average minimum temperature of the coldest month (°C)	Annual precipitation (mm)
Duchesnay	DUC	46.87	−71.67	250	Single	3.6	−34	1361.9
Lapocatière	LAP	47.36	−70.03	22	Multiple	4.5	−28.5	937.1
Shawinigan	SHW	46.53	−72.65	124	Multiple	4.7	−32.6	1057.7
First Eel Lake	FEL	45.83	−67.62	177	Single	5.1	−30.8	1158.7
Coy Brook	COB	46.27	−65.53	89	Single	5.5	−29.7	1124
Sherbrooke	SHR	45.38	−71.92	301	Multiple	5.5	−31.9	1136.7
Cantley	CAN	45.57	−75.78	154	Multiple	5.7	−31.7	999

A total of 1008 seedlings were grown in the forest nursery in Berthierville, QC, Canada. After germination in 2020, seedlings grew in an 85% peat, 7.5% vermiculite and 7.5% perlite substrate with added lime (9 kg lime per 3.1 m^3^ peat). Seedlings stayed in tunnels covered by transparent plastic until reaching 25 cm in height and were then transferred outdoors under a shading net. After entering dormancy, the seedlings spent the winter in a cold room at −3 °C. In May 2021, the seedlings were transplanted in trays containing 15 cavities of 320 cm^3^ and placed outdoors in two open sites, Chicoutimi and Ripon (QC, Canada, Figure 2). We chose the two sites for their location with respect to maple distribution. Chicoutimi (48°25′ N, 71°02′ W) and the surrounding region represent the northern limit of maple’s range (Godman et al. 1990). Ripon (45°46′ N, 75°06′ W) is a warmer site, 420 km southwest of Chicoutimi, located within the natural range of sugar maple (Table 2).

**Table 2:** Characteristics of the two sites where the seedlings were grown before sampling for the frost hardiness assessment. Climate data is relative to the 1980–2010 period (source: BioSIM).

Name	Latitude	Longitude	Elevation (m a.s.l.)	Annual temperature (°C)	Average minimum temperature of the coldest month (°C)	Extreme minimum temperature (°C)	Annual precipitation (mm)
Ripon	45.78	−75.10	180	4.9	−18.7	−43.3	1091
Chicoutimi	48.42	−71.05	82	3.1	−21.3	−43.3	931

### Climate and weather data

Climate and weather data were used to compare the provenances and understand the drivers of frost hardiness. We obtained historical (1980–2010) climate averages for the two sites of sampling and seven geographic provenances using BioSIM (Régnière et al. 2014). We also used BioSIM to obtain weather data for the period of measurement (winter 2021/2022). We obtained snow cover and historical daily temperatures from the nearest available weather station, i.e. Chénéville (13 km from Ripon) and Bagotville (9 km from Chicoutimi) (Environment Canada 2023). Growing degree days (GDD) were calculated from January 2022 onwards with a base threshold of 0 °C to track above-zero temperature accumulation during the spring. Cumulative chilling days were calculated for the acclimation period (September–January) to quantify cold accumulation in the two sites. Chilling units (CU) were calculated daily as CU = max(7 – T_m_; 0), where T_m_ is the mean daily temperature. We used cumulative daily CU instead of the more common chilling hours because hourly temperatures were missing for Ripon. The threshold of 7 °C was used as suggested in the literature for sugar maple (Weinberger 1967, Raulier and Bernier 2000).

### Frost hardiness tests

We performed monthly frost hardiness measurements from September 2021 to July 2022. Seedlings were 2-year-old at this time, having spent their second growing season outdoors at each site. We defined frost hardiness as the temperature inducing 50% of cellular damage (LT_50_), measured with the relative electrolyte leakage (REL) method (Repo and Lappi 1989). All provenances were sampled monthly except COB, which was sampled every 2 months because of a smaller number of seedlings. Overall, we sampled 154 seedlings per provenance and 84 seedlings for COB. All samples were analyzed in Chicoutimi. Samples from Ripon were delivered to the lab within 24 h of collection, and immediately started the REL analysis. To minimize the impact of the 24-h lag between sampling and analyses, samples were placed in plastic bags with wet paper towels to maintain humidity and prevent dehydration and were shipped in a Styrofoam box for insulation from external temperatures during transport.

On each sampling date, seven seedlings per provenance were collected in each site. Different samples were collected on each date. Seedlings were separated into three samples, each at least 5 cm long, and distributed randomly between seven target temperatures for the frost treatment. In total, each target temperature had three samples per provenance, wrapped in tin foil and placed in a thermal container. Two thermocouples per thermal container were used to keep track of temperature during the tests, one attached to a random sample and one measuring air temperature inside the container.

During each test, we exposed the samples to seven different treatment temperatures ranging from +5 to −80 °C. One thermal container was stored in a cold chamber at +5 °C (control treatment). A second thermal container was stored in a freezer at −80 °C for 4 h (lethal treatment) before being transferred to +5 °C. The remaining five thermal containers were exposed to five different temperatures ranging from −7 °C to −60 °C in a controlled-temperature freezer (EH40–2–3, Envirotronics, Grand Rapids, MI, USA). The target temperatures changed during the sampling dates to better quantify the expected frost hardiness, mostly with regular intervals of −10 to −15 °C between target temperatures (i.e. we tested colder temperatures during winter see Figure S1 available as Supplementary data at *Tree Physiology* Online). The temperature in the freezer was manually adjusted to attain a cooling rate of −10 K h^−1^. This rate is higher than the cooling rate of 5 K h^−1^ normally used in the literature and faster than freezing rates normally observed in nature (Cannell and Sheppard 1982, Atucha Zamkova et al. 2021). However, in our case, the temperature of the freezer needed to be continuously and manually adjusted in small temperature increments since the freezer model did not have a function for controlled temperature descent. This made a cooling rate of −5 K h^−1^ logistically unattainable, requiring 17 h of continuous presence and input to control the descent rate. Upon reaching one of the target temperatures, one random container was taken out of the freezer and stored in a cold chamber at +5 °C. All containers were then left at +5 °C overnight.

On the second day, the samples were prepared for conductivity measurements. Each seedling was separated into branches (cut in slices 0.5 mm thick) and buds (split in two along the longitudinal axis), then stored in vials with 10 mL of demineralized water. Because of the small number of buds available, buds from the same provenance and within the same target temperature were placed in the same vial. Vials were left to agitate on a multi-platform orbital shaker (Thermo Fisher Scientific, Waltham, MA, USA) at 5 °C overnight.

**Figure 3:**
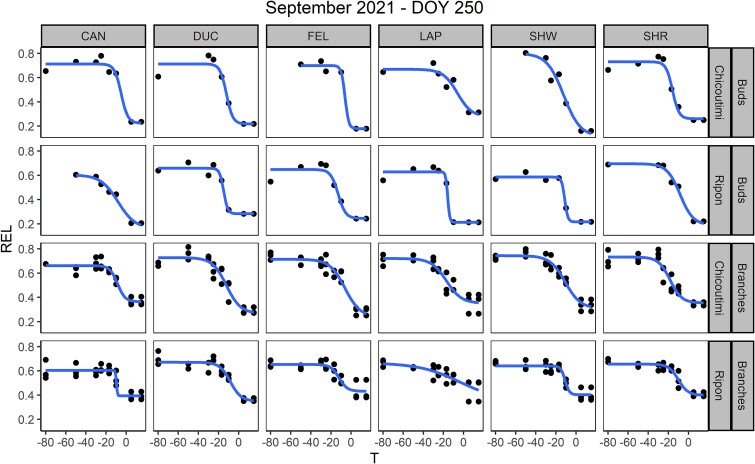
Example of the results of the REL analysis, divided by provenance (columns), organs and site of sampling (rows). Black dots are REL measurements at different test temperatures for each provenance and organ (branches, buds). Logistic curves (lines) were fitted to estimate LT_50_ based on REL measurements. L50 was estimated as the inflection point of the logistic curve.

The third day, conductivity in each tube was measured as an indicator of electrolyte leakage from cells damaged by the frost (C_1_). Samples were then put in an autoclave at 120 °C, 1.2 bar, for 30 min. A second conductivity measurement was performed after the autoclave treatment, corresponding to the maximum cellular damage (C_2_). REL was then calculated as C1/C2, i.e. the ratio of electrolyte leakage caused by frost to the leakage caused by maximum damage. We calculated the relationship between REL and temperature using the logistic function (Repo and Lappi 1989): 


(1) \begin{align*}\mathrm{REL}=\frac{a}{\left(1+{e}^{b\left(c-T\right)}\right)}+d \end{align*}

where *T* is the test temperature, *d* is the higher asymptote, *a + d* is the lower asymptote, and *b* is the slope at the inflection point *c*. Frost hardiness (LT_50_) was calculated for each provenance as the temperature at the inflection point *c*, i.e. the temperature causing 50% of cellular damage (Figure S2 available as Supplementary data at *Tree Physiology* Online). Temperatures inducing 10% cellular damage (LT_10_) were also calculated from the logistic curve to estimate a lower threshold for frost damage occurrence and for comparison with LT_50_. The logistic function was fitted with the nlsLM function of the minpack.lm package (Elzhov et al. 2022). An example of the end result of monthly REL analyses illustrating the logistic fittings used to estimate LT_50_ can be found in Figure 3.

### Statistical analyses

We applied Wilcoxon rank sum exact tests to compare the differences in frost hardiness between sites on specific sampling dates. Based on the observed pattern of frost hardiness, we identified three periods: (i) acclimation, corresponding to the increase in frost hardiness during autumn (between September and December), (ii) maximum hardiness, corresponding to the peak in frost hardiness during midwinter (between December and March) and (iii) deacclimation, corresponding to the decrease in frost hardiness during spring (between March and June). For each period, the differences in frost hardiness between sites and provenances were tested using ANCOVA. We set LT_50_ as the response variable, circadian days since the start of the experiment (7 September 2021) as quantitative covariate, and site and provenance as categorical variables. We fitted ANCOVA with the ANOVA function of the rstatix package (Kassambara 2023). We tested for normality and homoscedasticity with the Shapiro–Wilks and Levene’s tests, respectively. Model goodness-of-fit was evaluated by adjusted *R*^2^ values, distribution of standardized residuals and visual assessment of residuals plots.

We fitted a circular regression model to investigate differences between site and provenance during the whole frost hardiness cycle. Circular regressions allow recurrent biological events to be described by transforming the independent variable, i.e. time, into a circular variable expressed in radians (Batschelet 1981). We used circular regression in order to test for site (environment) and provenance effects with a model that could account for frost hardiness variation throughout the year. We expressed the time as circadian days since the start of the experiment, transformed into radians (t_rad_). The *sine* and *cosine* functions of t_rad_ represent the seasonal pattern of frost hardiness in the model. We fitted a circular model with LT_50_ as the response variable and the *sine* and *cosine* functions of t_rad_ as quantitative explanatory variables, in addition to the factors site (two levels) and provenance (seven levels). We used delta-AIC (Akaike Information Criterion) to compare models with and without the variables for site, provenance and their interaction in order to select the best model, both for ANCOVA and circular regression. Delta-AIC comparison was carried out with the *aictab* function of the AICcmodavg package (Mazerolle 2023). All statistical analyses were performed in R (R Core Team 2020).

## Results

### Weather conditions

**Figure 4:**
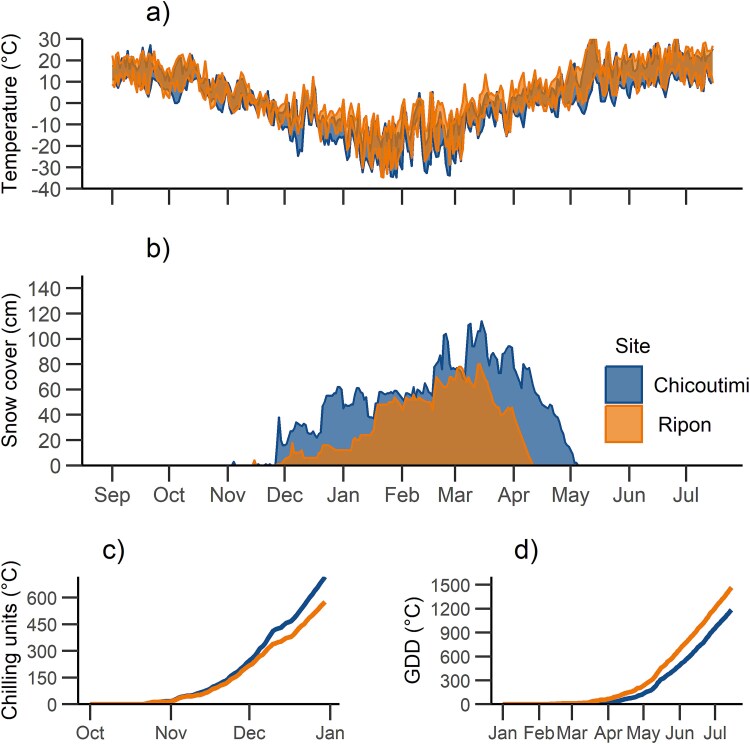
Trends of (a) air temperature (°C), (b) snow cover (cm), (c) daily CU (°C) and (d) growing degree-days (GDD °C) during the study period in winter 2021/2022. Air temperature is shown as a shaded area between daily maximum and minimum values. Data was obtained from the nearest weather stations (Environment Canada 2023).

From September 2021 to July 2022, the northern site (Chicoutimi) and southern site (Ripon) experienced a mean temperature of 0.4 and 3.1 °C, respectively. On average, the temperatures differed from 1 to 1.2 °C between sites during the autumn (September–November), with Ripon being the warmer site. Chilling units’ accumulation, i.e. the days with mean daily temperature <7 °C, started on day of the year (DOY) 295 (22 October) in both sites and reached 16 and 18 degree-days in Ripon and Chicoutimi, respectively on DOY 305 (1 November). The difference in CU between sites remained below 50 until DOY 341 (7 December), then increased to 144 on DOY 365 (31 December), with Chicoutimi being the coldest site (Figure 4). In winter (December to February) Chicoutimi was colder by an average of 4.7, 4.3 and 3.9 °C for minimum, mean and maximum temperatures, respectively. The lowest daily minimum temperature recorded during winter was −34.7 °C on both sites, which occurred on DOY 22 in the southern site (Ripon) and on DOY 29 in the northern site (Chicoutimi). In spring (March to June), Chicoutimi was 2.5–2.8 °C colder than Ripon. On DOY 121 (1 May), the GDD above 0 °C reached 233 in Ripon and 134 in Chicoutimi. Snow cover appeared on the same date in the two sites, on DOY 331 (27 November) (Figure 4, lower panel), reaching a height of 114 cm in Chicoutimi, 34 cm more than in Ripon. The snow on the soil disappeared on DOY 100 (10 April) in Ripon, 24 days earlier than in Chicoutimi (Figure 4).

### Acclimation

**Figure 5:**
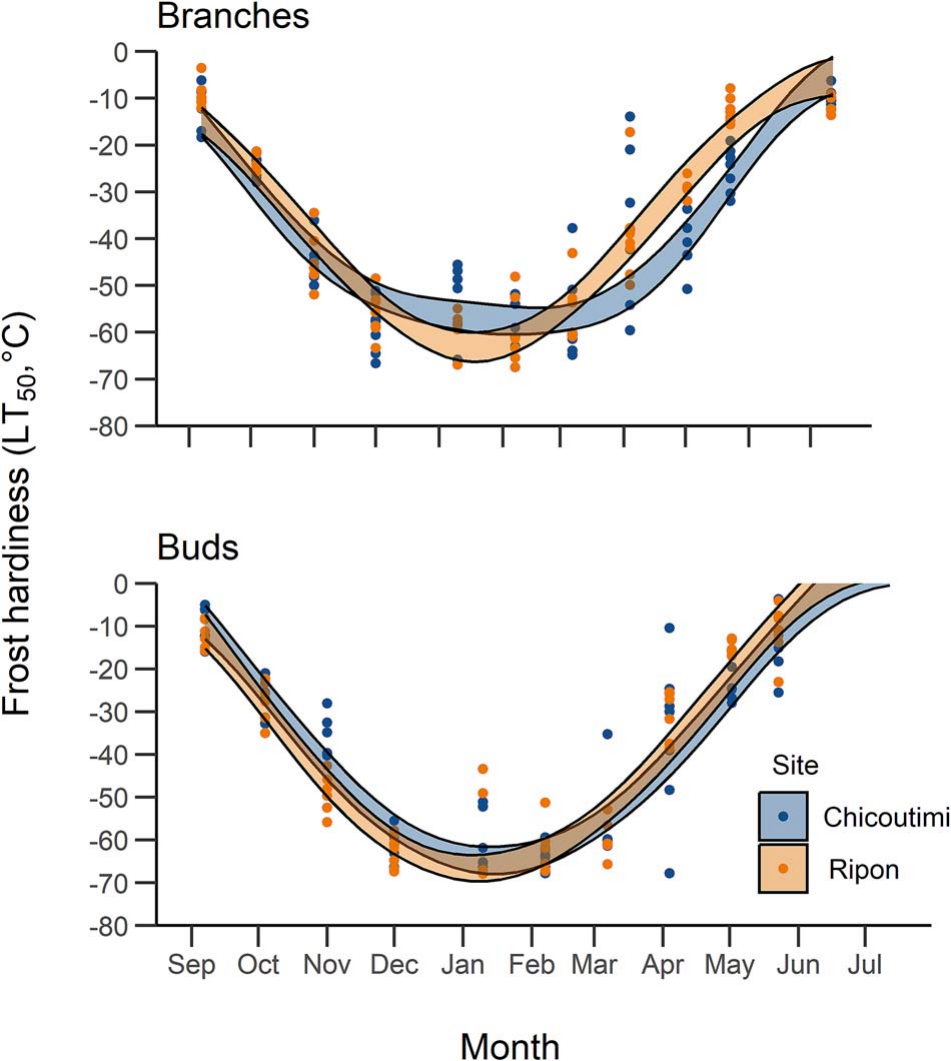
Frost hardiness (LT_50_) of seven sugar maple provenances sampled in two study sites for branches (above) and buds (below). Points indicate estimated values of LT_50_. Shaded areas indicate the 95% confidence interval of the circular model’s prediction. Ripon is the southern site, and Chicoutimi the northern one.

On average, LT_50_ of branches in early September was −12 and −9 °C in Chicoutimi and Ripon, respectively (Figure 5). At the same time, bud LT_50_ was −9 and −12 °C in Chicoutimi and Ripon, respectively. From September to December, LT_50_ decreased by an average of 15 °C per month in both organs. In early December, LT_50_ reached −58 °C in branches and −62 °C in buds in Chicoutimi. At the same time, LT_50_ in Ripon were −55 °C and −62 °C for branches and buds, respectively (Figure 5). The overall lowest frost hardiness for branches was assessed in early September, −6 °C in Chicoutimi (provenance FEL) and −3.5 °C in Ripon (provenance LAP). LT_50_ in buds was significantly higher in Chicoutimi (−35.9 ± 5 °C) than in Ripon (−49 ± 4.7 °C) only in November, according to the Wilcoxon test (W = 36, *P* < 0.01, Table 3). LT_50_ in branches was not significantly different between sites. The analysis of covariance (ANCOVA) for acclimation was significant, showing an effect of site on LT_50_ in buds (*P* < 0.05) but not in branches. The effect of provenance during acclimation was not significant (Table 4).

**Table 3:** Results of the Wilcoxon bilateral rank sum exact test comparing differences in LT_50_ between sites for different organs and sampling dates. One, two and three asterisks correspond to *P* < 0.05, *P* < 0.01 and *P* < 0.001, respectively.

Organ	Date (dd-mm-yyyy)	*W*
Buds	07-09-2021	24
	04-10-2021	24
	01-11-2021	36^**^
	01-12-2021	24
	10-01-2022	24
	01-02-2022	20
	07-03-2022	22
	04-04-2022	22
	02-05-2022	3^*^
	23-05-2022	20
Branches	07-09-2021	11
	04-10-2021	24
	01-11-2021	16
	01-12-2021	19
	10-01-2022	27
	07-02-2022	37
	07-03-2022	12
	04-04-2022	26
	02-05-2022	0^**^
	23-05-2022	0^***^
	11-07-2022	39

**Table 4:** ANCOVA results testing the effect of site and provenance during the periods of acclimation (September–December), maximum hardiness (December–February) and deacclimation (March–July). The variable time indicates days since the start of the experiment. The adjusted *R*_2_ for goodness of fit is shown with F values and significance levels for the whole model and terms. One and three asterisks correspond to *P* < 0.05 and *P* < 0.001, respectively.

		Model		Terms			
		R^2^	F	(Intercept)	Time	Site	Provenance
Branches	Acclimation	0.93	88.95^***^	63.69^***^	702.96^***^	1.95	0.19
	Maximum hardiness	0.02	1.113	230^***^	0.01	0.81	1.34
	Deacclimation	0.85	35.78^***^	425.97^***^	254.57^***^	9.12^***^	0.91
Buds	Acclimation	0.93	89.04^***^	29.39^***^	699.77^***^	5.65^*^	0.37
	Maximum hardiness	−0.05	0.6982	300.75^***^	1.77	0.05	0.63
	Deacclimation	0.88	33.64^***^	358.78^***^	259.00^***^	0.68	0.38

### Maximum hardiness

From December to early March, LT_50_ reached a plateau and fluctuated around low temperatures (Figure 5). During this period, the variation in LT_50_ between monthly sampling dates was, on average, below 4 °C in branches and below 5 °C in buds. Between December and March, the lowest LT_50_ attained in Chicoutimi were −58 °C in branches and −64 °C in buds), which were measured in December and February, respectively. In Ripon, the lowest LT_50_ were −60 °C in branches and −63 °C in buds, measured in February and December, respectively (Figure 5). Among provenances, the provenance SHW reached the lowest values of LT_50_ in Chicoutimi, −68 °C for branches in December and −67 °C for buds in February. In Ripon, the lowest LT_50_ was measured on SHW for buds (−68 °C in January) and on COB for branches (−67 °C in February). According to the Wilcoxon test, there were no significant differences between sites during this period (Table 3). ANCOVA for this period was not significant, and with low R^2^ (Table 4). This was due to the lack of variability in LT_50_, which made the inclusion of explanatory variables in the model redundant.

### Deacclimation

From March to May, LT_50_ for branches in Ripon rose from −55 °C to −13 °C, with an average increase of 14 °C per month, declining to 1 °C per month between late May and July. LT_50_ for buds in Ripon increased from −59 °C to −15 °C between March and early May, a rate of 16 °C per month, which decreased to 4 °C per month between early and late May. In Chicoutimi, LT_50_ for branches increased from −57 °C to −9 °C between March and July (11 °C per month). Between March and May, LT_50_ for buds in Chicoutimi rose from −56 °C to −13 °C, with an average increase of 14 °C per month (Figure 5). LT_50_ in April showed the highest variability among sampling dates. This was likely due to the high conductivity of the sugar-rich sap that maples produce during spring reactivation (Perkins and van den Berg 2009), which altered the REL. For this reason, LT_50_ from April was considered unreliable and excluded from the successive analyses (i.e. ANCOVA and circular regression analyses).

The overall lowest frost hardiness in buds was measured in late May, −3.6 °C in Chicoutimi (provenance DUC) and −4 °C in Ripon (provenance SHR). Frost hardiness was significantly lower in Ripon in early May, according to Wilcoxon test (W = 3, *P* < 0.05, Table 3). Frost hardiness in branches was significantly lower in Ripon on both sampling dates of May (W = 0, *P* < 0.01), with Ripon showing the lower frost hardiness (Figure 5). ANCOVA indicated a significant effect of site on LT_50_ in branches (*P* < 0.01), but not in buds. Provenance had no significant effect on LT_50_ for the two organs (Table 4).

### Circular model

Delta AIC-based model selection indicated that the best circular model for LT_50_ included the terms *cos* and *sin*, which define the time, and site as a fixed effect, which represented two different environments and their interaction. The provenance was discarded during model selection for both branches and buds.

In the final circular model for branches, LT_50_ changed mainly as a function of time (*cos* and *sin* variables and their interaction, *P* < 0.001, Table 5). This indicates the strong seasonality of frost hardiness, with an increase in autumn (acclimation) and a decrease in spring (deacclimation), as described earlier. The site also had a significant effect on LT_50_ in branches (*P* < 0.01, Table 5). The interactions between *sin* and *cos* function, as well as their interaction with site, were significant (*P* < 0.05, Table 5), indicating that the site influences the shape of the circular regression curve, i.e. the rate of acclimation and deacclimation. In the final circular model for buds, *cos*, *sin* and their interaction were significant (*P* < 0.05, Table 5), indicating the seasonal pattern of LT_50_ following acclimation and deacclimation. Site was not significant but had a significant interaction with the *sin* function (*P* < 0.1, Table 5).

**Table 5:** Results of circular modeling of frost hardiness (LT_50_) in sugar maple branches and buds. LT_50_ is modeled as a function of time (*cos* and *sin* variables), sampling site and provenance. The operator ‘×’ is used to indicate interactions between variables. The adjusted *R*_2_ for goodness of fit is shown with the significance level of the linear regression. *t* values are shown with the significance level of single terms. Points correspond to *P* < 0.1. One, two and three asterisks correspond to *P* < 0.05, *P* < 0.01 and *P* < 0.001, respectively.

Branches					
*R* ^2^	*F*	Terms	Estimate	St. error	*t*
0.90	171.1^***^	(Intercept)	−35.88	0.78	−46.07^***^
		*cos*	20.63	1.16	17.86^***^
		*sin*	−17.15	1.09	−15.81^***^
		site	2.18	1.10	1.98^*^
		*cos* × *sin*	−11.19	2.25	−4.98^***^
		*cos* × site	−1.65	1.63	−1.01
		*sin* × site	−4.70	1.53	−3.06^**^
		*cos* × *sin* × site	12.23	3.18	3.84^***^
Buds					
*R* ^2^	*F*	Terms	Estimate	St. error	*t*
0.91	195.9^***^	(Intercept)	−31.59	1.00	−31.50^***^
		*cos*	22.55	1.44	15.69^***^
		*sin*	−27.30	1.46	−18.75^***^
		site	−0.11	1.30	−0.09
		*cos* × *sin*	−4.52	2.51	−1.80
		*sin* × site	−3.39	1.87	−1.81
		*cos* × site	−2.03	1.77	−1.15

## Discussion

Frost hardiness ranged between −4 °C during the growing season and −68 °C in the coldest period of winter, showing the typical pattern of acclimation, lower plateau and deacclimation reported in the literature for other species. This range of values confirms the capacity of sugar maple to maximize its frost resistance during the dormant period (Sakai and Larcher 1987, Charrier et al. 2015). Our results show that acclimation (i.e. increasing frost hardiness levels) took place between September and December. LT_50_ reached a plateau between December and March, followed by deacclimation (i.e. decreasing frost hardiness) until July.

Differences between sites were observed mainly during the deacclimation in spring, with the southern site showing a faster decrease in frost hardiness (Figure 5), corresponding with the earlier warming recorded at that site (Figure 4). No difference in LT_50_ was detected between provenances. Our results suggest that frost hardiness adaptations are similar across the sampled area, located in the northern portion of sugar maple range. Local environment had greater importance than geographic provenance in determining the dynamics of frost hardiness, in agreement with previous studies reported in the literature (Charrier et al. 2011, 2018).

### Acclimation

Overall, acclimation in branches was similar between sites, contradicting our hypothesis that seedlings in the colder site would acclimate faster. This lack of difference is likely due to the similar weather and photoperiod in the two sites. Chilling accumulation was similar between October and early December 2021, with daily temperatures differing on average by 1 °C between sites. Moreover, the two sites have similar photoperiods, with differences during the acclimation period ranging between 10 min on 1 September and 20 min on 21 December. Temperature and photoperiod are the main environmental signals, inducing growth cessation, dormancy and cold acclimation (Weiser 1970, Vitasse et al. 2014*b*, Charrier et al. 2015). Therefore, similar weather conditions can explain the converging acclimation patterns between the two sites.

Buds showed more differences between sites during acclimation, as shown by the significant effect of site on frost hardiness (Table 4). Although LT_50_ was similar on most sampling dates, differences in LT_50_ were detected in November, with the southern site (Ripon) showing higher frost hardiness. The mean temperatures during the week before sampling were warmer in the southern site (7.3 °C) than the northern site (6.2 °C), which is counterintuitive since we expected a higher frost hardiness in the colder site. The observed difference in LT_50_ may be due to the sampling day (see section 0). In the northern site, samples were collected on 1 November, after a short warm event with minimum temperatures reaching 3.6 °C. In the southern site, samples were collected on 2 November, after a cold night with minimum temperatures falling to −1 °C. The frost could have stimulated a rapid adjustment of frost hardiness in the southern site, which would explain the differences observed. Several other studies documented quick fluctuations of tree frost hardiness in response to temperature changes during dormancy (Sakai and Larcher 1987, Neuner et al. 1999, Vitasse et al. 2014*b*), which confirms our hypothesis.

### Maximum hardiness

During the coldest part of the winter (between January and February) frost hardiness reached stable and maximum values, with LT_50_ varying between −43 and −68 °C. All provenances attained LT_50_ below −55 °C in both sites. The low R^2^ of ANCOVA indicated a lack of trend in LT_50_, which varied around maximum values without a distinct pattern (Table 4). Our LT_50_ values are much lower compared with those recorded in other temperate deciduous species, which in most cases reach maximum values of −40 °C (Charrier et al. 2011, Vitra et al. 2017). This could reflect the harsher conditions of our sampling sites, which lie close to the border between the temperate and boreal forest. Indeed, a study on wild blueberry (*Vaccinium* spp.) conducted in the same geographical area as our study found LT_50_ of −68 °C during the winter, which are similar to our findings in maple (Deslauriers et al. 2021). Cold hardiness below −60 °C, and occasionally below −70 °C, is known for several boreal woody species (Sakai 1983, Sakai and Larcher 1987).

In our experiment, provenances growing in the colder site (Chicoutimi), were effectively moved to the northern limit of the species (Godman et al. 1990). LT_50_ in Chicoutimi exceeded both the lowest daily minimum temperature recorded during our study (−34.7 °C), the average minimum winter temperature of the last 30 years (from 1990 to 2020) (−33.4 ± 2.7 °C) and the lowest minimum temperature recorded since 1981 (−43.3 °C; Environment Canada 2023). Even when considering the more conservative LT_10_ (i.e. temperature inducing 10% of cellular damage), all provenances attained values below −40 °C, indicating a good ability to minimize frost damages in harsh winter conditions. Our results demonstrate that Canadian sugar maple provenances transferred to northern areas at the limit of the species range can endure the minimum temperatures expected under winter conditions. This is consistent with a 3-year common garden study on sugar maple seedlings, which found that northern populations are able to survive and grow well beyond the species’ northern distribution limit (Putnam and Reich 2017). Several other studies have found that deciduous species generally exhibit a frost hardiness higher than the local minimum temperatures of winter (Lenz et al. 2013, Kollas et al. 2014, Charrier et al. 2018).

Extreme frost events could limit the expansion and establishment of sugar maple. Extreme events are difficult to predict (Jentsch et al. 2007) and can be particularly relevant when considering trees, which have a long life span, particularly in the northern regions. An extreme frost could exceed the frost hardiness of maple, cause widespread mortality and have long-lasting effects on a developing stand (Montwé et al. 2018). For example, a single extreme frost event occurring more than 30 years after the date of planting induced a widespread mortality in southern provenances of *Pinus pinaster* (Ait.) in southern France, highlighting a maladaptation that was previously not evident (Benito-Garzón et al. 2013).

In our experimental design, the exposure of samples to target temperatures did not last more than a few minutes. This means that our LT_50_ corresponded to the temperature that would cause instantaneous lethal damage. It is possible that longer exposures (e.g. several hours during a nighttime frost) to low temperatures above the observed LT_50_ could cause severe or lethal damage to maple. Because of the inherent differences between controlled experiments and natural conditions, the link between LT_50_ or different levels of damage and actual mortality still remains to be quantified (Burr et al. 2001). In our protocol, we used 10 K h^−1^ as a freezing rate, while the thawing rates (i.e. the rate of warming of the samples after the frost treatments) were not controlled. In the literature, most studies adopt freeze rates of 5 K h^−1^, which seems to be closer to what observed during freezing events in the field (Atucha Zamkova et al. 2021). Fast freeze or thaw cycles can increase frost damages; therefore, caution should be applied when comparing our results with LT_50_ obtained with different protocols.

### Deacclimation

Deacclimation generally lasted from March to late May for all organs and sites, with the exception of branches in the northern site (Chicoutimi), which continued to lose frost hardiness between the end of May and beginning of July. Differences between sites were evident in May, with the warmer southern site showing an earlier and faster deacclimation. These results point to a strong environmental control over deacclimation, consistently with our hypothesis. It is well known that environmental signals, such as temperature and photoperiod, have a strong influence on ecodormancy release and budbreak (Hänninen 1997, Kovaleski et al. 2018). Temperature is regarded as the main factor inducing ecodormancy release and budbreak (Junttila 2007, Charrier et al. 2015), while the effect of photoperiod is less linear than temperature and is often species-specific (Hänninen and Tanino 2011, Flynn and Wolkovich 2018). In our study, warmer temperatures in the southern site led to faster snowmelt and GDD accumulation (Figure 4). This suggests a strong effect of temperature, a likely explanation for the observed differences in LT_50_ between sites. Photoperiod, on the other hand, was longer in the northern site after the equinox (20 March, DOY 79). A longer photoperiod would have induced a faster deacclimation in the northern site, contrary to what was observed in our case. It is also possible that differences in photoperiod between sites (30 min at maximum) were too marginal to affect the experiment. We conclude that deacclimation in sugar maple is mainly driven by temperature in our study region, which is consistent with other studies on spring reactivation and budbreak in this species (Guo et al. 2020, Ren et al. 2020).

Further research should focus specifically on investigating intra-specific variations in spring late frost risk. Our results indicate that sugar maple is sensitive to warm spring temperatures, which could lead to early budbreak and frost damage in the case of false spring events (Chamberlain et al. 2019). Moreover, there is evidence of intra-specific differences in budbreak phenology for sugar maple in the literature (Guo et al. 2020, Zeng et al. 2022). This is an important aspect to consider since the risk of desynchronization between spring phenology and favorable environmental conditions is increased under climate change (Augspurger 2013).

### Intra-specific differences

Our experimental results found no significant differences in frost hardiness between provenances. The provenance factor variable was not significant in ANCOVA when performed separately for acclimation and deacclimation. Moreover, model selection for the circular regression considering the whole frost hardiness cycle discarded the provenance factor. Most of the variation in LT_50_ is explained by the time of sampling, with site having a minor effect. This lack of intra-specific differentiation may stem from the limited geographic gradient considered in our study. We selected seven provenances from the same geographic area, i.e. southern Quebec and New Brunswick. This corresponds to the northern distribution of sugar maples, which extends southwards to Tennessee (US) and westwards to the states of Missouri and Minnesota (Godman et al. 1990). Recent unpublished data on sugar maple genetic diversity showed no clear genetic structure within this study area, with the larger variation occurring between individuals than between provenances, thus demonstrating a high level of gene flow (2024 unpublished data by Y. Surget-Groba; unreferenced), Kriebel (1957) studied the intra-specific variation of ecophysiological traits in sugar maple across a wider range and proposed three broad ecotypes: southern, central and northern. Similarly, a study of maple spring phenology by Buttò et al. (2023) found intra-specific differences only when considering the whole species range. It is therefore likely that the provenances considered here are genetically homogeneous and have similar adaptations, belonging to the northern ecotype. Other studies considering a wider geographical gradient could find higher intra-specific variability.

In the current study, we do not assess the risk of off-season frost, i.e. frost events occurring before acclimation in autumn (early frost) or after deacclimation in spring (late frost). However, within their distribution range, there is evidence that sugar maple provenances differ in their spring phenology, likely because of local adaptations to avoid late frost risk (Guo et al. 2020, Zeng et al. 2022). To study these critical stages with more accuracy, the meteorological gradient should be enlarged to include warmer conditions simulating the expected future temperatures in the northern range of the distribution. Moreover, future investigations should consider observations at higher temporal resolutions (weekly), in order to include differences in phenology between the provenances. Such an approach might test whether spring phenology and deacclimation dynamics are more important drivers of tree species range limits than winter frost hardiness, as suggested in previous studies (Kollas et al. 2014, Körner et al. 2016). Further studies are needed to quantify the chilling and forcing requirements regulating dormancy dynamics in sugar maple provenances (Chuine et al. 2016). This aspect is particularly important for forest management and provenance selection, since late frost damage can affect tree establishment and growth during the first years after planting (Hufkens et al. 2012, Vitasse et al. 2014*a*).

## Conclusions

This study assessed frost hardiness in sugar maple seedlings belonging to seven Canadian provenances and growing in two sites in Quebec, at the northern portion of the species’ range. Over the 2021/2022 dormant season, frost hardiness was similar between sites during the periods of acclimation and maximum hardiness. This contradicted our hypothesis that seedlings in the northern sites would have faster acclimation and higher frost hardiness during winter. Deacclimation was faster and earlier in the southern and warmer site, partially confirming our hypothesis and underlining the importance of temperature in determining the timings of budbreak.

We did not find any significant difference between provenances in either acclimation, maximum hardiness period or deacclimation. LT_50_ was far lower than the long-term minimum temperatures occurring at the northern border of the species range. This suggests that winter conditions are not a limiting factor for the northward expansion of sugar maple, consistently with existing knowledge in the literature (Lenz et al. 2013, Kollas et al. 2014, Putnam and Reich 2017, Charrier et al. 2018). In order to minimize the risks of frost for maple, the climatic characteristics of the planting site should have priority in respect to provenance selection. Provenance selection should be guided by other factors, such as phenological avoidance of late frosts, growth performance and enhancing biodiversity and gene flows between populations (Aitken and Bemmels 2016). This information is particularly relevant in forest management projects considering the northward transfer of provenances, i.e., assisted migration (Pedlar et al. 2011).

## Supplementary Material

Supplementary_material_tpae167

## Data Availability

The original data and R codes that support the findings of this study are available on request from the corresponding author.
