# Supplementary material for: Local conditions have greater influence than provenance on sugar maple (Acer saccharum Marsh.) frost hardiness at its northern range limit
Source: Tree Physiol. 2024 Dec 27;45(1):tpae167. doi: 10.1093/treephys/tpae167 (PMC11761971; doi:10.1093/treephys/tpae167)

# Supplementary material

For the manuscript “Local conditions have greater influence than provenance on sugar maple (Acer saccharum Marsh.) frost hardiness at its northern range limit.”

**Figure S** 1: Target temperatures tested in Relative Electrolyte Leakage (REL) analysis by date of sampling.


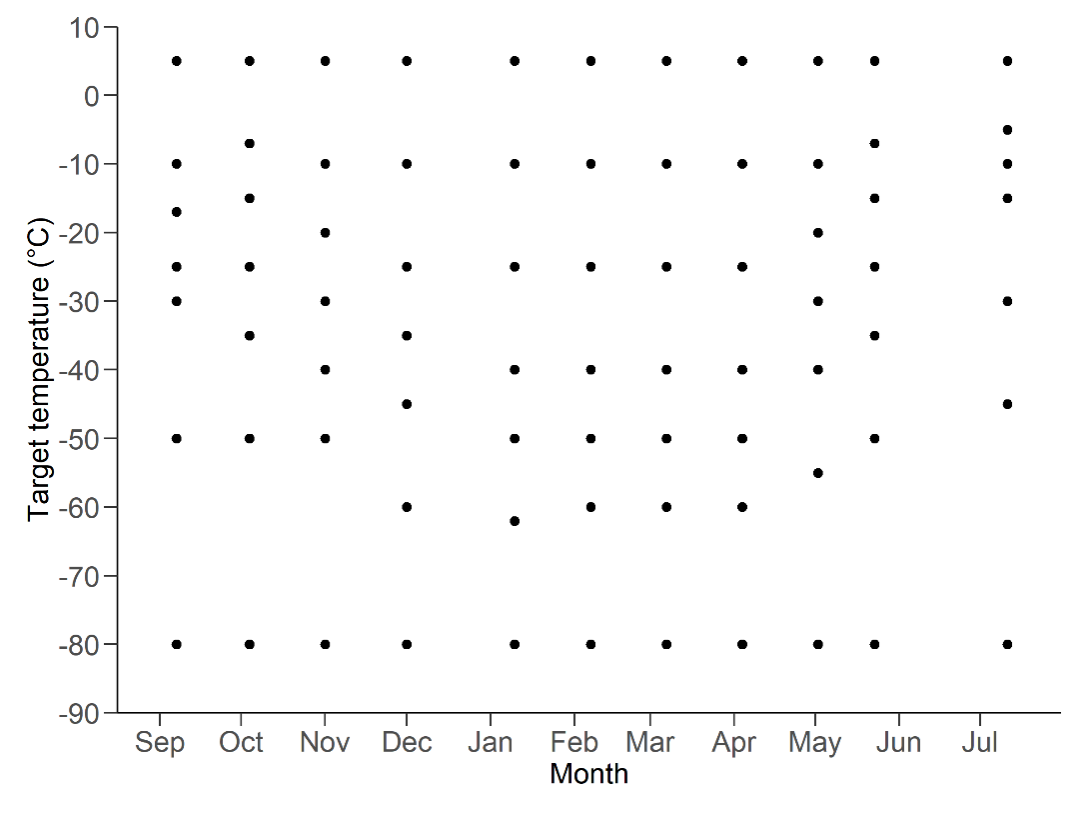


**Figure S**2 (continued): Logistic curves (in blue) fitted to estimate LT50 based on Relative Electrolyte Leakage (REL) measurements. Black dots are REL measurements at different test temperatures of sampling for each provenance and organ (branches, buds). L50 was estimated as the inflection point of the logistic curve.


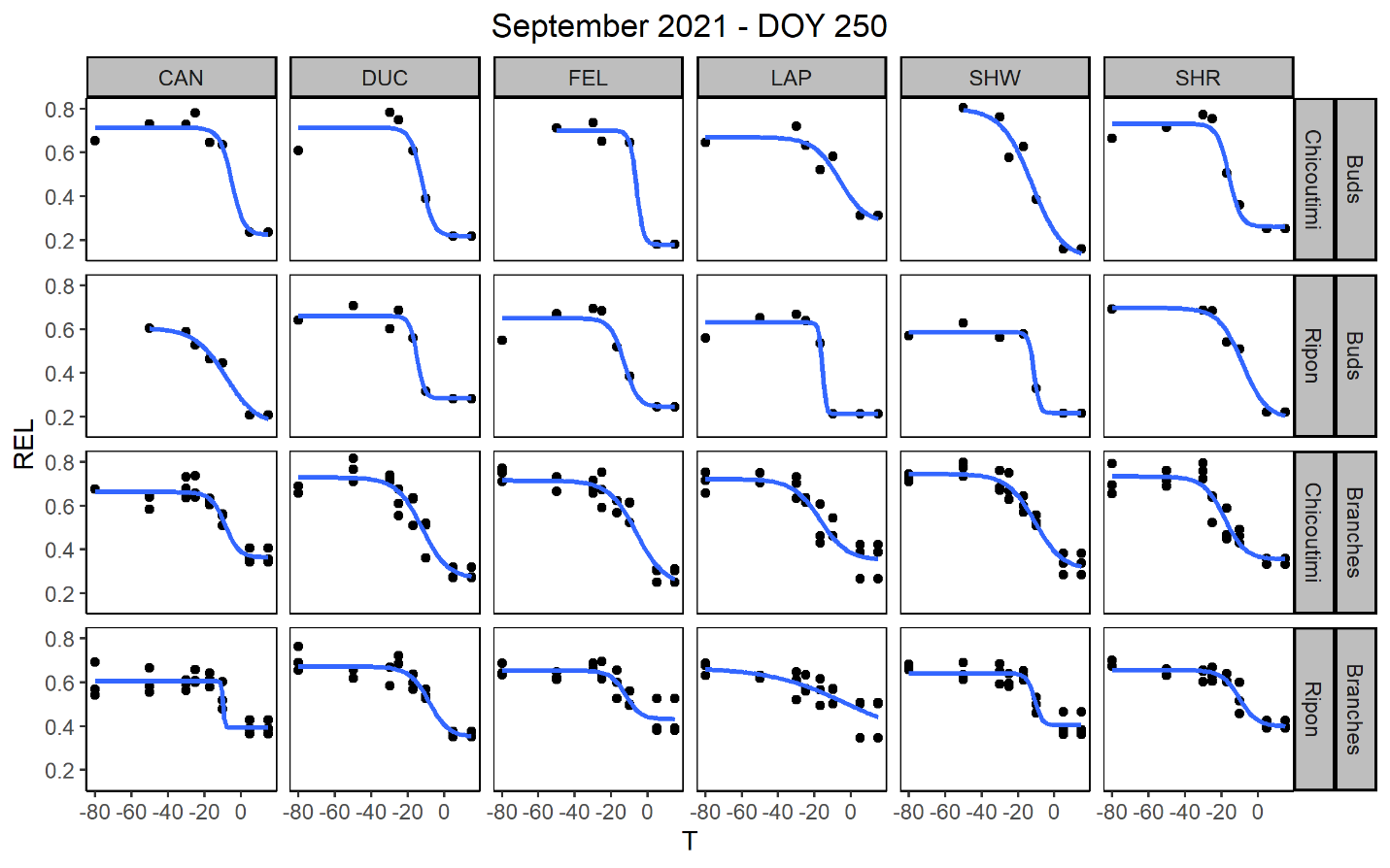


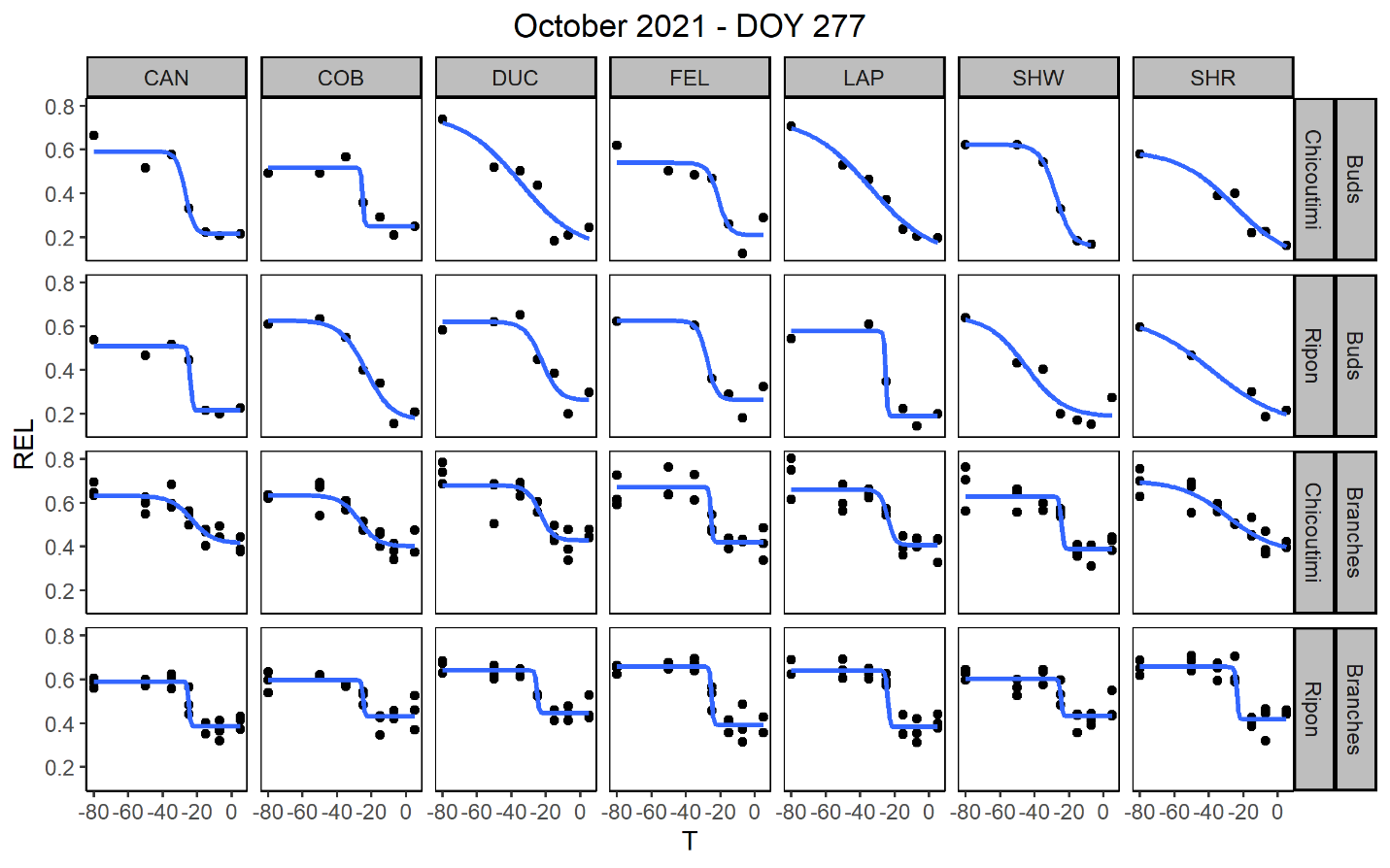


Figure S 2 (continued)


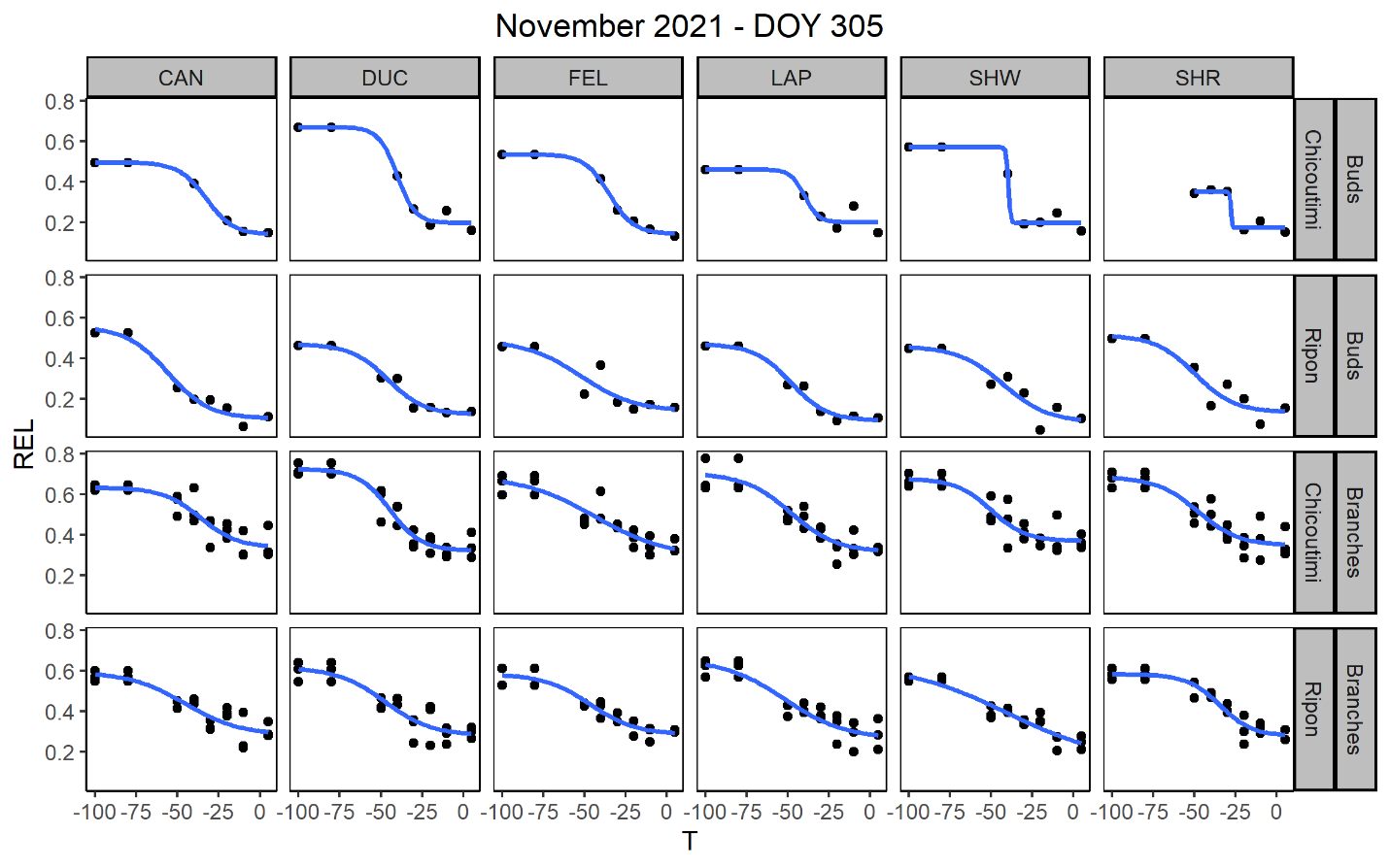


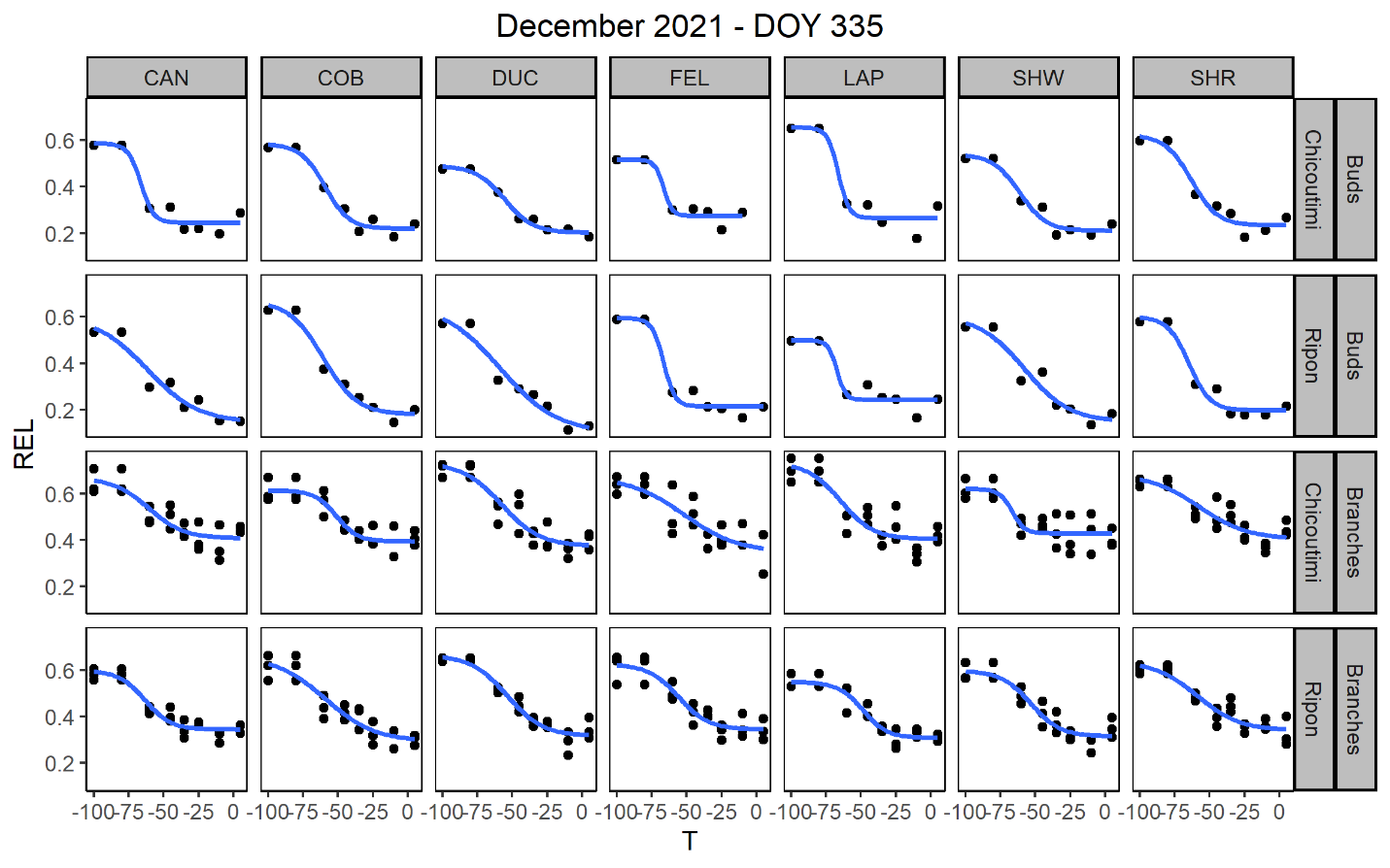


Figure S 2 (continued)


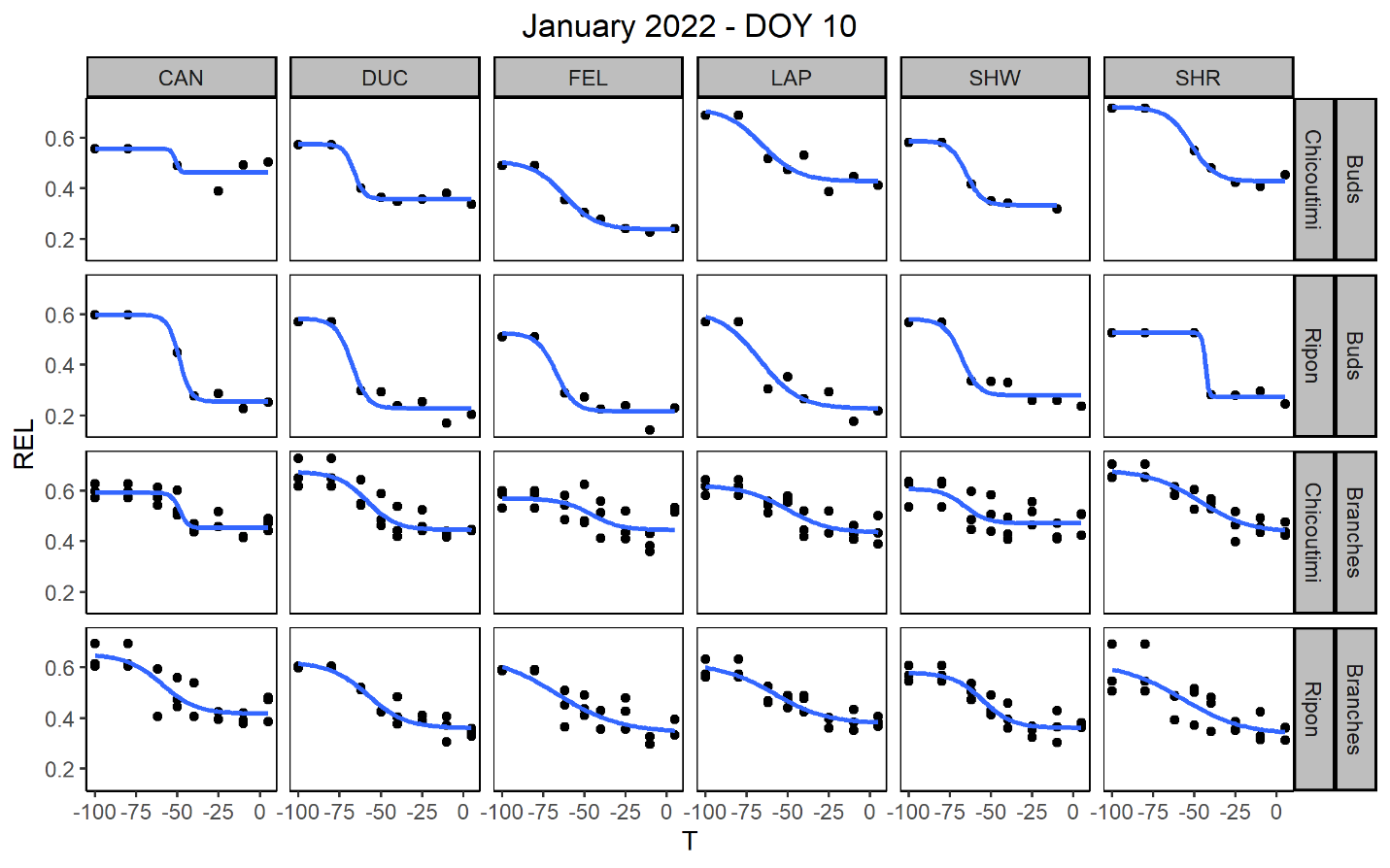


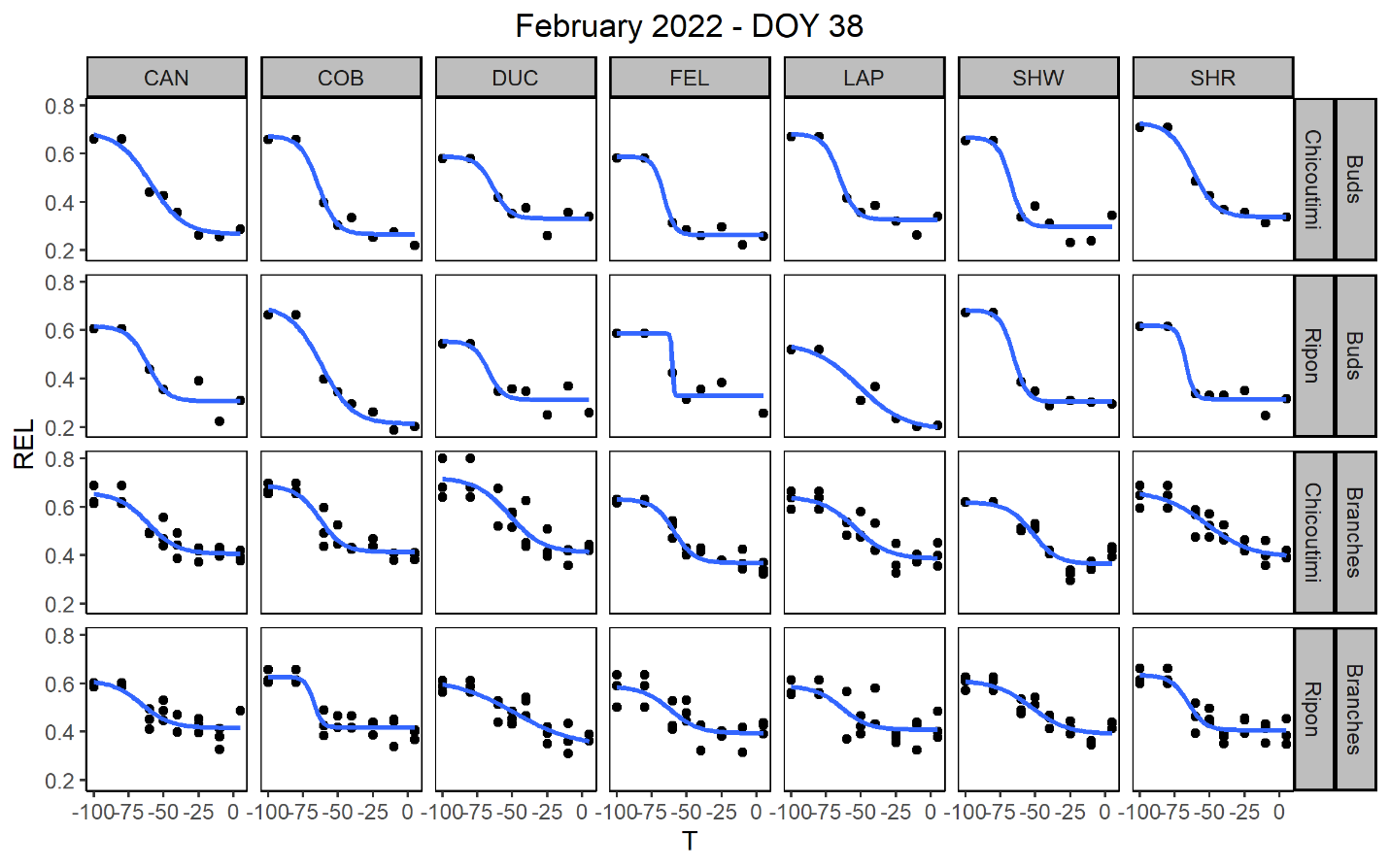


Figure S 2 (continued)


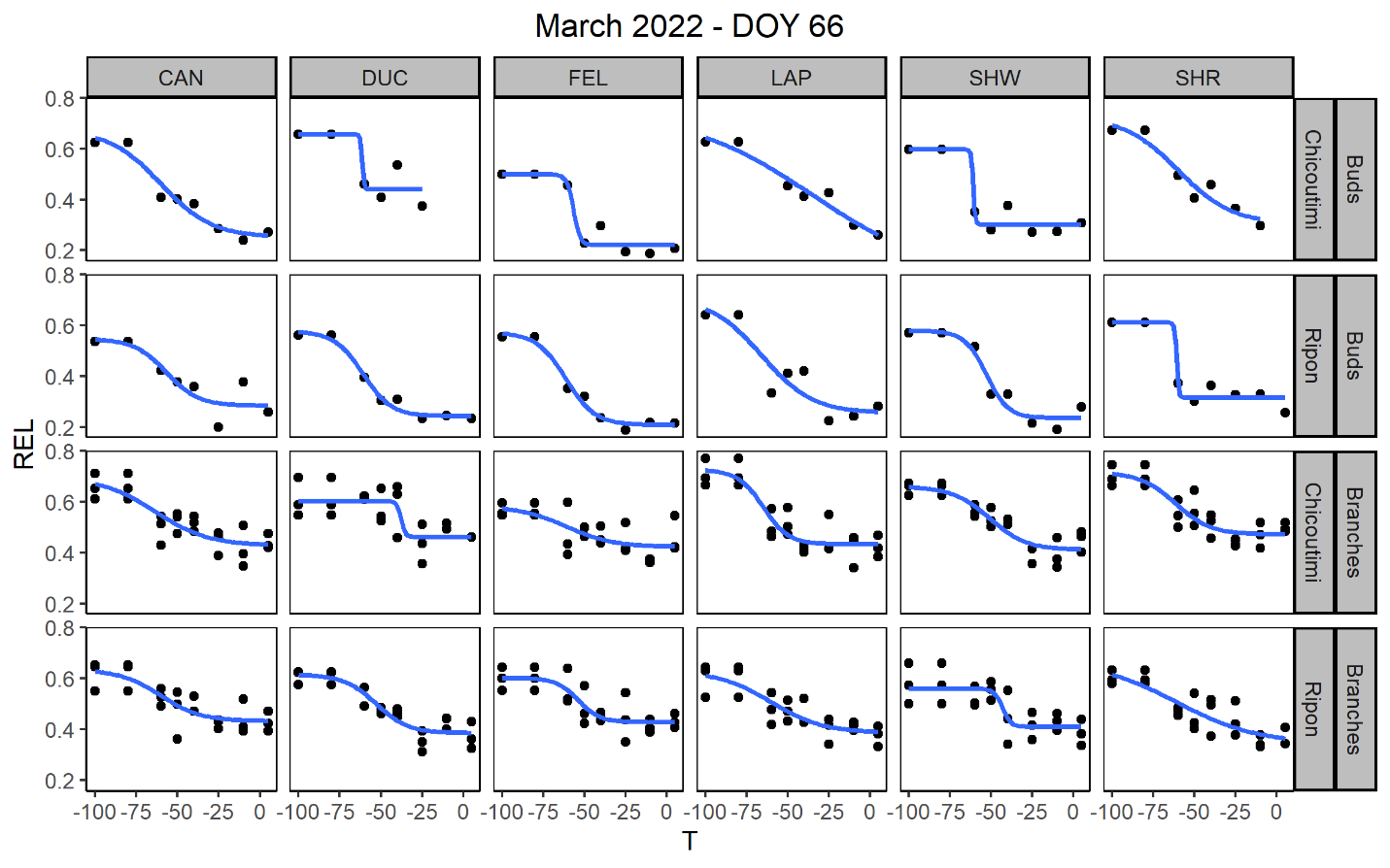


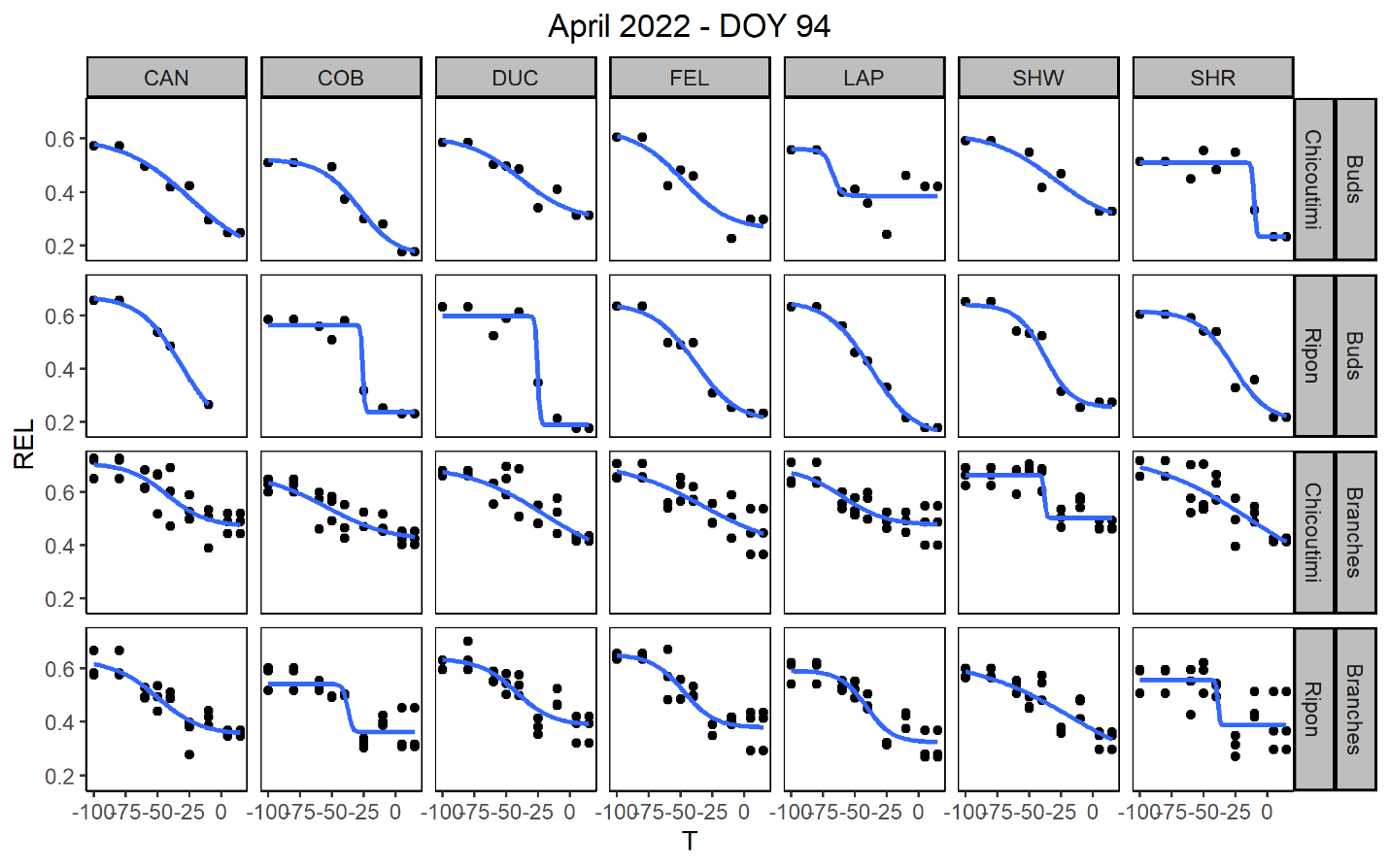


Figure S 2 (continued)


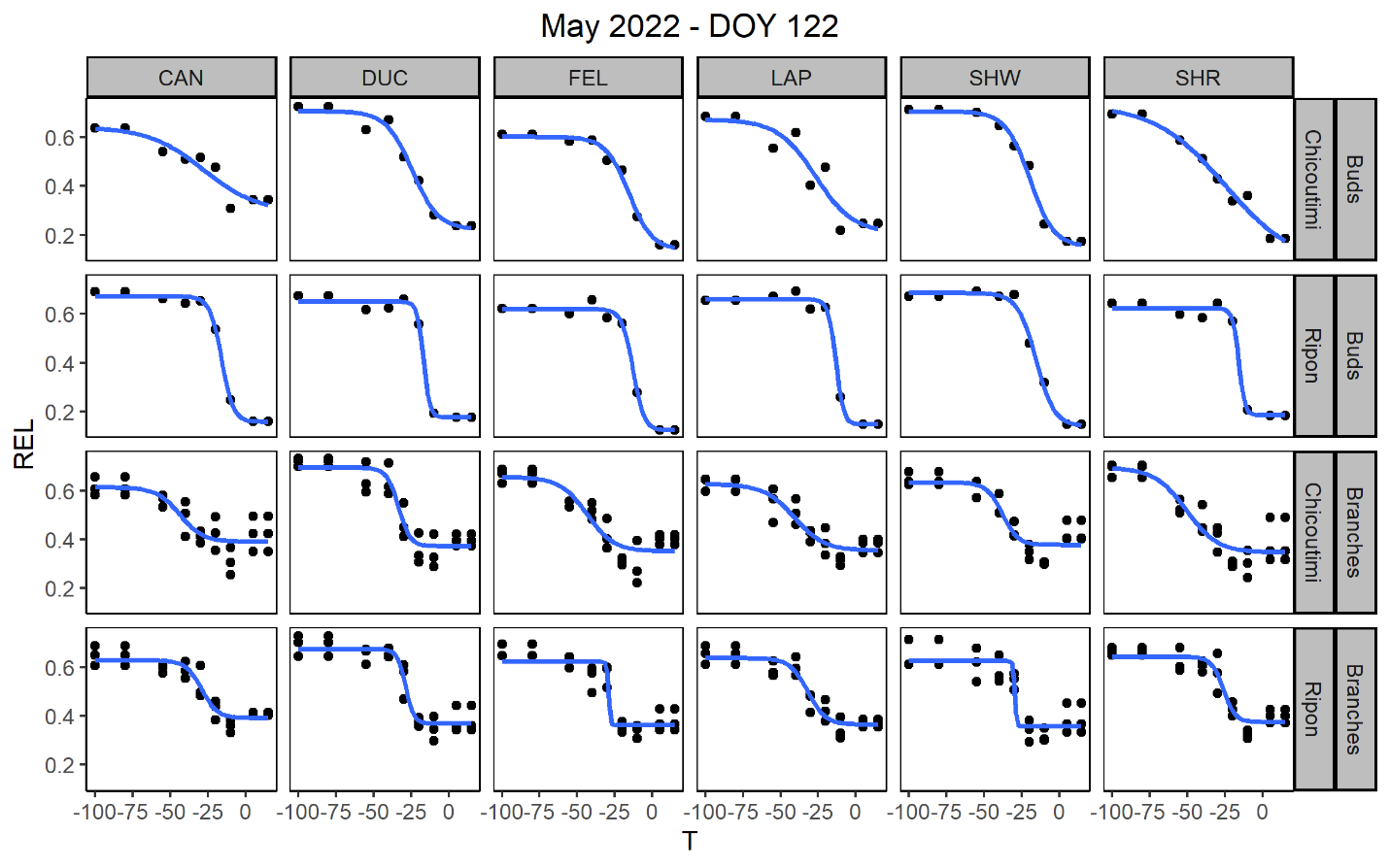


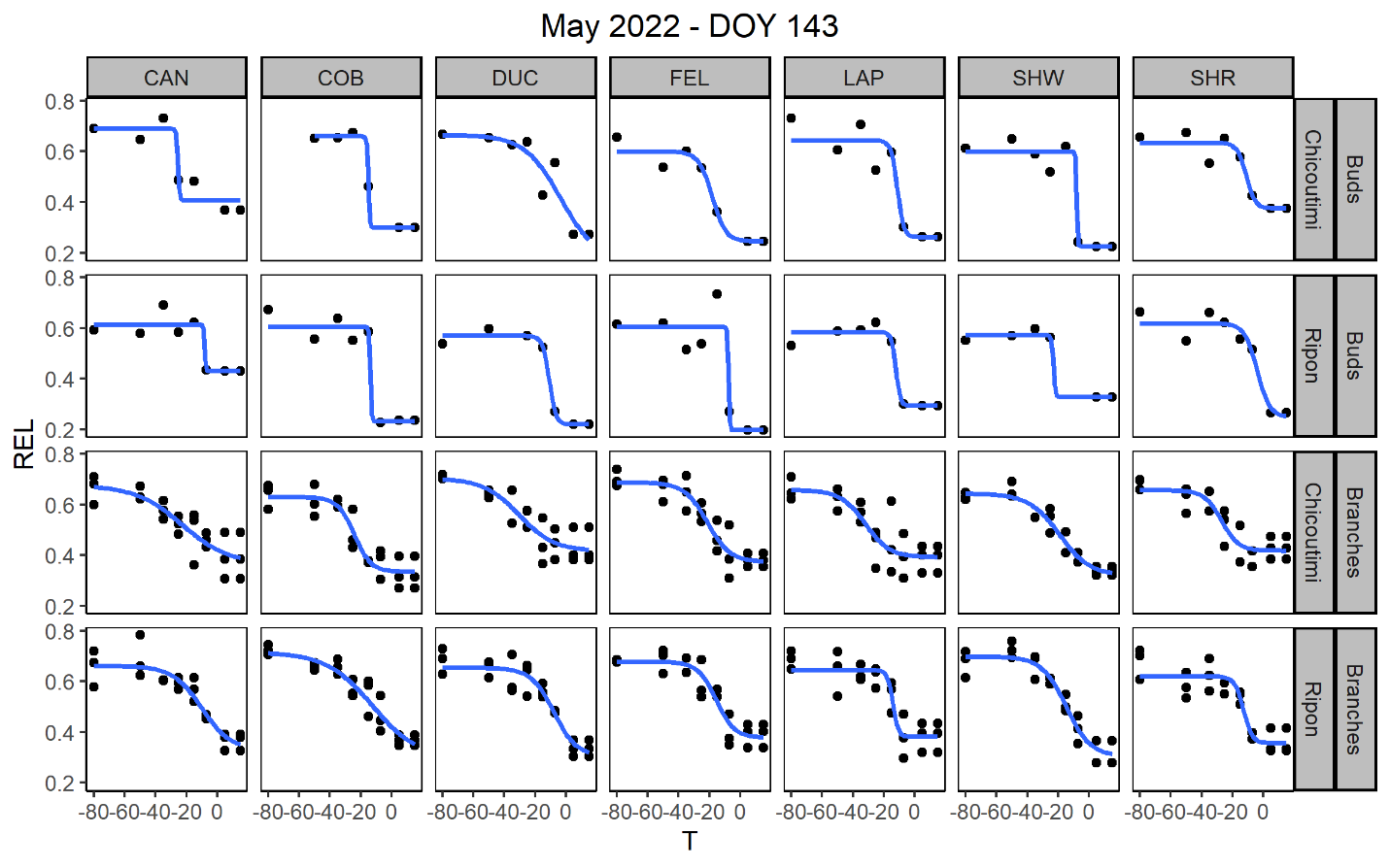


Figure S 2 (continued)


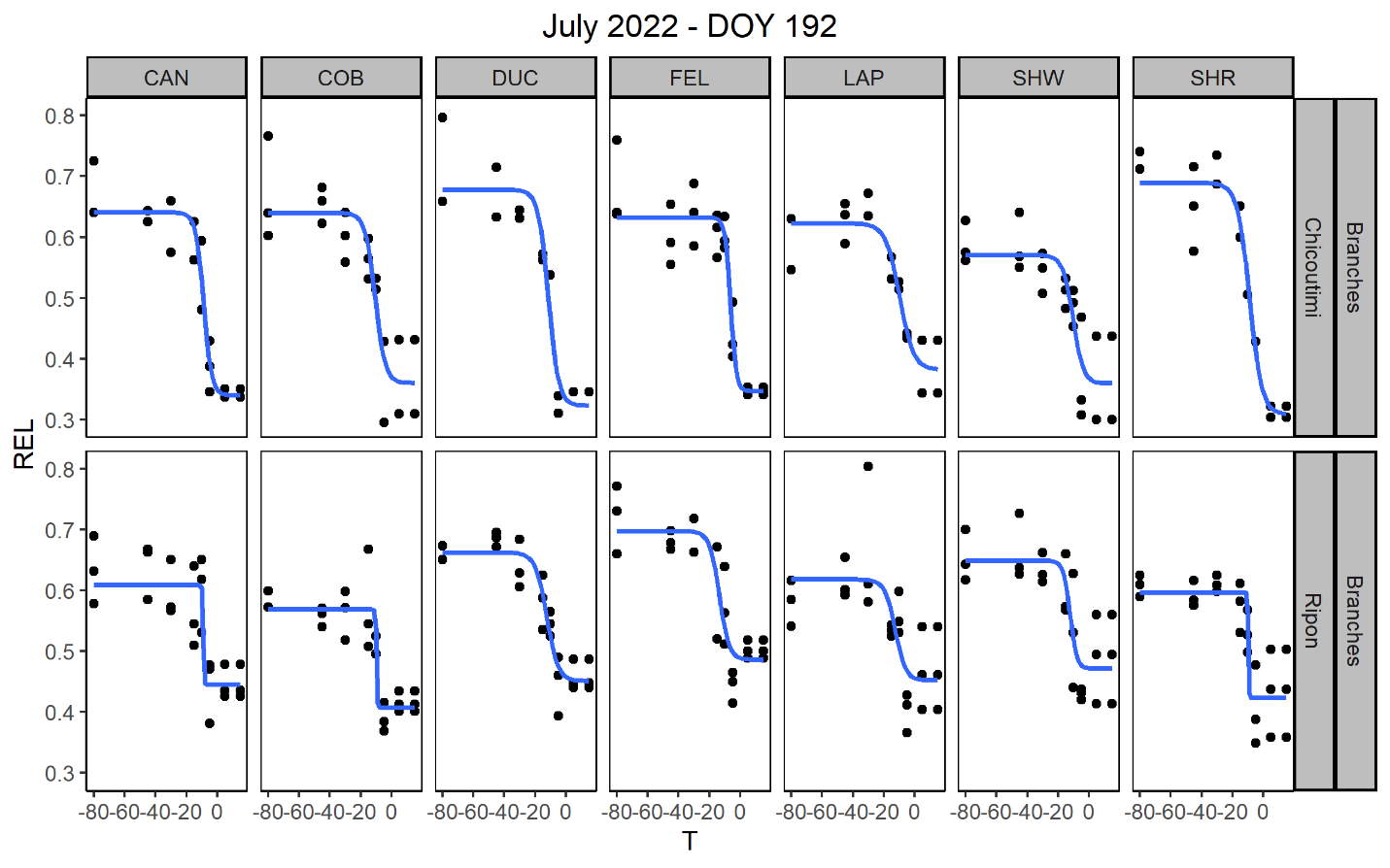

Supplement: Supplementary_material_tpae167 [file supplementary_material_tpae167.docx]
